# An Analysis of Natural T Cell Responses to Predicted Tumor Neoepitopes

**DOI:** 10.3389/fimmu.2017.01566

**Published:** 2017-11-15

**Authors:** Anne-Mette Bjerregaard, Morten Nielsen, Vanessa Jurtz, Carolina M. Barra, Sine Reker Hadrup, Zoltan Szallasi, Aron Charles Eklund

**Affiliations:** ^1^Department of Bio and Health Informatics, Technical University of Denmark, Kongens Lyngby, Denmark; ^2^Instituto de Investigaciones Biotecnológicas, Universidad Nacional de San Martín, Buenos Aires, Argentina; ^3^Section for Immunology and Vaccinology, National Veterinary Institute, Technical University of Denmark, Kongens Lyngby, Denmark; ^4^Computational Health Informatics Program (CHIP), Boston Children’s Hospital, Harvard Medical School, Boston, MA, United States

**Keywords:** neoepitopes, neoantigens, prediction, immunogenicity, mutations, MHC binding

## Abstract

Personalization of cancer immunotherapies such as therapeutic vaccines and adoptive T-cell therapy may benefit from efficient identification and targeting of patient-specific neoepitopes. However, current neoepitope prediction methods based on sequencing and predictions of epitope processing and presentation result in a low rate of validation, suggesting that the determinants of peptide immunogenicity are not well understood. We gathered published data on human neopeptides originating from single amino acid substitutions for which T cell reactivity had been experimentally tested, including both immunogenic and non-immunogenic neopeptides. Out of 1,948 neopeptide-HLA (human leukocyte antigen) combinations from 13 publications, 53 were reported to elicit a T cell response. From these data, we found an enrichment for responses among peptides of length 9. Even though the peptides had been pre-selected based on presumed likelihood of being immunogenic, we found using NetMHCpan-4.0 that immunogenic neopeptides were predicted to bind significantly more strongly to HLA compared to non-immunogenic peptides. Investigation of the HLA binding strength of the immunogenic peptides revealed that the vast majority (96%) shared very strong predicted binding to HLA and that the binding strength was comparable to that observed for pathogen-derived epitopes. Finally, we found that neopeptide dissimilarity to self is a predictor of immunogenicity in situations where neo- and normal peptides share comparable predicted binding strength. In conclusion, these results suggest new strategies for prioritization of mutated peptides, but new data will be needed to confirm their value.

## Introduction

Tumor cells can be naturally recognized by the adaptive immune system based on the sequence and abundance of the immunogenic peptides presented on the tumor cell surface. The majority of known tumor antigens are either normal peptides expressed at an unusually high level, or peptides derived from translation of somatic mutations (neoepitopes).

Neoepitopes are important in several successful approaches to enhance tumor killing by T cells. Inhibitors of immune checkpoints such as programmed cell death protein 1 and cytotoxic T lymphocyte-associated protein 4 counter the inhibition of T cell responses often observed in cancer patients. With both of these approaches, a higher mutation load predicts greater clinical benefit, suggesting that neoepitopes are important for immune response, at least in selected cancer types (1, 2). Adoptive transfer of expanded tumor-infiltrating lymphocytes (TILs) increases the proportion of tumor-responsive T cells, and selective expansion of neoepitope-specific T cells has been successfully demonstrated (3). Recent studies using peptide vaccines of patient-specific neoepitopes have shown that targeting neoepitopes may be an effective method to treat cancer (4, 5). Thus, rapid and accurate identification of patient-specific neoepitopes is an important goal.

Neoepitopes can be identified in various ways. Early studies used T cell reactivity screening of cDNA expression libraries (6). More recently, a common approach is to first identify somatic mutations by sequencing DNA and/or RNA from a tumor and matched normal specimen. These somatic mutations can be used to infer changes in protein sequences, resulting in “neopeptides” that are potentially present in tumor cells but not in normal cells. Such candidate neopeptides can next be synthesized and tested for reactivity by autologous T cells using various assays such as ELISPOT, fluorescently labeled HLA tetramers, or barcode-labeled HLA multimers (7). However, most neopeptides do not serve as neoepitopes. For a neopeptide to become a neoepitope, at least two properties must be fulfilled: the peptide must be processed and presented by HLA, and the presented peptide must be recognized by a suitable T cell. The problem of predicting neoepitopes can, therefore, be split into two individual problems: (1) predict neopeptide antigen processing and presentation (presentation) and (2) predict which peptides, if presented by HLA, can trigger a T cell response (immunogenicity).

Predicting HLA presentation is typically done based on prediction of HLA binding between an individual peptide sequence and the relevant HLA alleles, using tools such as NetMHC (8) or NetMHCpan (9). If available, mRNA expression data may be used to eliminate neopeptides from genes that are not expressed. Abelin et al. (10) trained a prediction algorithm considering mass spectrometry (MS) data from eluted peptides, peptide expression and cleavage, outperforming NetMHC 4.0 and NetMHCpan 2.8, although this predictor is not yet publicly available. It should be mentioned that the newest version (4.0) of NetMHCpan is also trained on MS eluted ligand data as well as binding affinity data (11).

To predict immunogenicity, one proposal has been to use the “differential agretopic index” (DAI), defined as the difference in binding strength between the mutated neopeptide and its unmutated normal peptide counterpart (12). The reasoning behind this is based on the mechanism of immune tolerance ensuring that no T cell will recognize HLA presented self peptides. Given this, one way for a neopeptide to become a neoepitope would be to have significantly improved HLA-binding capacity compared to the normal peptide. In this situation, only the neopeptide will be presented by surface HLA, and hence, no tolerization is present against the normal peptide. Consequently, tolerization against the neopeptide is expected to play a minor role for the immunogenicity in this situation. In contrast, when the neopeptide and the normal peptide are both HLA binders, tolerization against the normal peptide has taken place, and this tolerization is expected to impact the immunogenicity of the neopeptide. Consequently, in this situation, the immunogenicity will depend on the lack of similarity between the mutated neopeptide and the normal counterpart.

Typically, only a minority of the tested neopeptides evoke a T cell response, suggesting that current methods to select candidate neopeptides are insufficient. In order to characterize the properties of neoepitopes, at least two groups have analyzed the characteristics of combined lists of published, confirmed neoepitopes. Van Buuren et al. (13) compiled a list of 17 neoepitopes that were identified without predictions and thus tested in an “unbiased” manner and found that their prediction algorithm would have correctly predicted 12 of the 17, for a sensitivity of 70%. However, this analysis does not provide a specificity estimate, and the data set is too small to analyze the relative importance of their individual selection criteria. Fritsch et al. (14) did a similar study on a larger group of 40 published neoepitopes, and found that in the majority of cases both the mutated and unmutated peptide were predicted to bind to HLA. Importantly, neither of these studies analyzed their data considering the set of neopeptides that did *not* elicit a T cell response. Recently, several studies have assessed immunogenicity of larger sets of neopeptides and have published lists of neopeptides, which both did and did not elicit a T cell response. We set out to analyze these data to investigate if any broad patterns emerge that might enable better predictions of neoepitopes.

## Materials and Methods

### Data Collection and Correction

Data was gathered from the 13 published papers (Table 1). For 10 of 13 studies, both neopeptides and the corresponding unmutated peptide were provided. For the other three studies, the corresponding normal peptides were missing or partially missing. For the missing normal peptides, we used “pepmatch,” a program available as part of MuPeXI (15), to identify the most similar peptide from the normal human peptidome. The normal human peptidome was defined as all unique peptides of lengths 8–11 extracted from human proteins available in Ensembl release 85, based on human genome GRCh38. Out of 820 neopeptides analyzed with pepmatch, 20 matched a reference peptide exactly with no mismatches, and 14 matched a reference peptide with more than a single mismatch. Additionally, one study included peptides originating from indels resulting in one peptide originating from a frameshift mutation being tested. An additional 7 peptide-HLA combinations were duplicates and were removed from the dataset together with the 35 non-single nucleotide variant (SNV) peptides; we note that none of these elicited an immune response. Thus, the final dataset included 1,948 peptide-MHC combinations of 27 HLA alleles and 1,874 unique mutated peptides. It should be noted that we included all 11 peptides from the study by Strønen et al., only two of these were found in autologous TILs, whereas the rest of the immunogenic peptides were identified in peptide stimulated PBMCs from healthy donors.

**Table 1:** Data included in this study.

Reference	Publication date	First and last author	Journal	Tumor type	Patients	Peptides tested	T cell responses	Test method	Peptide lengths
(16)	2013–05	Robbins et al. and Rosenberg	Nat Med	SKCM	3	227	10	ELISPOT	9–10
(17)	2013–11	Van Roij et al. and Schumacher	J Clin Oncol	SKCM	1	–	1	FLT	9
(18)	2014–03	Wick et al. and Nelson	Clin Cancer Res	HGSC	3	109	1	ELISPOT	8–11
(19)	2014–06	Rajasagi et al. and Wu	Blood	CLL	2	48	3	ELISPOT	9–10
(20)	2014–07	Lu et al. and Robbins	Clin Cancer Res	SKCM	2	10	2	ELISA	8–11
(1)	2014–12	Snyder et al. and Chan	N Engl J Med	SKCM	1	–	1	ICS	9
(2)	2015–04	Rizvi et al. and Chan	Science	NSCLC	1	–	1	FLT	9
(21)	2015–10	Cohen et al. Robbins	J Clin Invest	SKCM	8	427	9	FLT	9–10
(22)	2016–01	Kalaora et al. and Samuels	Oncotarget	SKCM	1	2	1	ICS	9, 11
(23)	2016–03	McGranahan et al. and Swanton	Science	NSCLC	2^a^	642^a^	3/8^a^	FLT/BLM	9–11
(24)	2016–05	Strønen et al. and Schumacher	Science	SKCM	4	56	11	FLT	9–11
(25)	2016–05	Bassani-Sternberg et al. and Krackhardt	Nature Commun	SKCM	1	8	2	MS-FLT	8–10, 12
(26)	2016–08	Bentzen et al. and Hadrup	Nat Biotechnol	NSCLC	2^a^	703^a^	9^a^	BLM	9–11

	Total	13		4	30	1,874	53	5	5

*1,874 unique tested peptides, 1,948 peptide-HLA combinations, from 27 HLA alleles*.

*FLT, fluorescently labeled tetramers; BLM, DNA barcode-labeled multimers; ICS, intracellular cytokine staining; MS, mass spectrometry; SKCM, skin cutaneous melanoma; NSCLC, non-small cell lung cancer; CLL, chronic lymphocytic leukemia; HGSC, ovarian high grade serous carcinoma*.

*^a^Peptides and patient overlap between studies*.

### Binding Prediction

We used a custom Python script to extract additional information, including the amino acid change, peptide mutation position, and number of mismatches, in addition to running NetMHCpan-4.0 (11) for HLA binding prediction and extracting the output predicted affinity and eluted ligand likelihood percentile rank (EL%Rank) score. NetMHCpan 4.0 is trained on both *in vitro* binding affinity and MS eluted ligand data and includes distinct prediction modes for each of the two data types. The default mode for NetMHCpan-4.0 (and the mode recommended for eluted ligand and epitope prediction) is eluted ligand-likelihood predictions. However, the user has the possibility to use the binding affinity mode by selecting the −BA. In this study, the recommended mode was used, evaluating the peptides on EL%Rank score. This score indirectly accounts for peptide cleavage and translocation when predicting peptide binding, as part of the dataset used for training consisted of MS identified HLA eluted ligands.

### Anchor Mutation Annotation

Anchor positions for each HLA allele were manually defined from NetMHCpan-3.0 sequence motifs (9). Peptides were annotated according to whether the mutation occurred in the given HLA allele anchor position (Table S1 in Supplementary Material, column: “BindingPosition,” “Anchor”).

### Analysis and Statistics

The resulting data was analyzed in R, and plots were generated using R packages ggplot2 and ggbeeswarm. *P-*values for difference in proportion were calculated using a two-sided Fisher’s exact test and/or Student’s *t-*test.

### Self-Similarity Predictions

The similarity between pairs of neo- and normal peptides was calculated using the kernel similarity measure proposed by Wen-Jun Shen et al. (27). The measure gives a value between 0 and 1 for the similarity of two peptides, where a value of 1 indicates a perfect match. In basic terms, this similarity is calculated from matching, at different length scales, all kmers (a substring of length *k*) in one peptide to the kmers in the other peptide using a Blosum similarity measure. In Figure S1 in Supplementary Material, we show the average similarity between a set of 9-mer peptide pairs with single mutations at different positions, forming 3,420 single mutant peptide pairs (20 random natural peptides each mutated to 19 single mutant variants at each of the 9 peptide positions). From this plot, it is clear that single mutation variations toward the N and C terminal of the peptide have very limited impact on the similarity between two peptides (the similarity is high) compared to mutations in the central part of the peptides (where the similarity is lower).

### Receiver Operator Characteristic (ROC) Curve Generation

Generally, a ROC curve is created by plotting the true positive fraction (or sensitivity) against the false positive fraction (or 1—specificity) at various threshold settings. In the ideal case, where a threshold exists that can perfectly separate the positive and negative data point, the area under the ROC curve (AUC) is 1, and in the situation where the predictive model has no predictive power, the ROC curve forms a straight line *x* = *y* and the AUC is 0.5. The plots where generated in R using the packages ggplot2 and plotROC.

The full dataset including all predictions, deselected peptides, HLA alleles, and additional peptide-specific information can be found in Table S1 in Supplementary Material.

## Results

We searched for published studies in which putative neoepitopes were first identified by tumor DNA sequencing and then experimentally tested for T cell reactivity. We chose to focus on studies in which native T cell reactivity against the minimal peptide of a neoepitope was assessed and did not include vaccine studies in which an induced T cell response was assessed. We considered only neopeptides derived from a SNV/missense mutation. We identified 13 relevant studies from which we created a dataset consisting of 1,948 unique peptide-HLA complexes, of which, 53 were reported to elicit a T cell response (Table 1; Table S1 in Supplementary Material). This represents 1,874 unique peptides; some of which were evaluated in combination with more than one HLA allele.

First, we searched for broad trends in factors that might influence neopeptide immunogenicity (Table 2). We analyzed the proportion of neopeptides eliciting a response according to neopeptide length and found that 9-mers had the highest relative frequency of response (4.3%), substantially higher than 10-mers (2.4%, *P* = 0.063) or 11-mers (0.2%, *P* = 0.00001). Note that this analysis accounts for the larger number of 9 and 10mer peptides experimentally evaluated compared to 8 or 11mer (Table 2). We next compared the three HLA genes and did not find a statistically significant difference between HLA-A and HLA-B (*P* = 0.50). The number of HLA-C restricted responses was too low to make any meaningful analyses related to the relative importance of this locus.

**Table 2:** Associations between peptide characteristics and T cell responsiveness.

	T cell response		Total	Proportion responding	*P*
	No	Yes			
**Peptide length**					
8mer	24	1	25	0.040	1.00
9mer	742	33	775	0.043	N/A
10mer	720	18	738	0.024	0.063
11mer	408	1	409	0.002	0.00001
12mer	1	0	1	0.000	1.00

**HLA gene**					
HLA-A	1,440	42	1,482	0.028	N/A
HLA-B	414	9	423	0.021	0.50
HLA-C	41	2	43	0.047	0.35

*P-values represent a test for difference in proportion responding, between the given row and the corresponding most frequent row (9mers or HLA-A)*.

**Figure 1:**
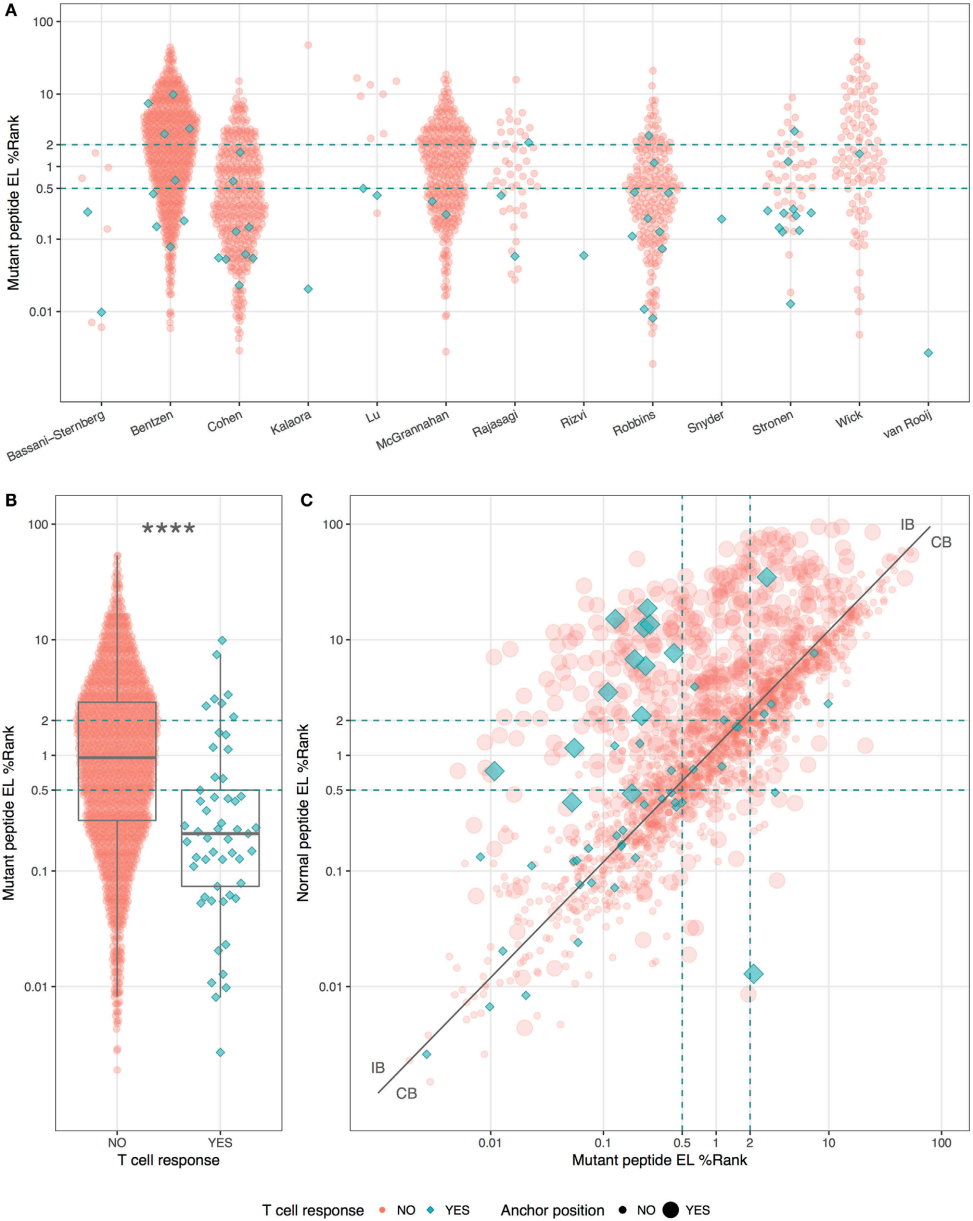
HLA-binding properties of neopeptides. Predicted eluted ligand likelihood percentile rank (EL%Rank) score of neopeptides, corresponding to individual studies **(A)** or summarized according to mutant peptide T cell response **(B)**. As there is an overlap in patients between the Bentzen and the McGrannahan study, only unique observations are plotted, the first refereeing to the peptides tested with barcode labeled multimers and the second with fluorescently labeled tetramers **(A)**. **(C)** Predicted EL%Rank score for neopeptides and their corresponding normal peptides, with mutant peptide T cell response and anchor position mutations indicated. The curve corresponding to the median EL%Rank_n_/EL%Rank_m_ value equal to 1.2, used to define the groups of peptides with improved binding strength (IB) and conserved binding strength (CB), is shown as a solid line. Thresholds for weak (2 EL%Rank) and strong binders (0.5 EL%Rank) are indicated with dashed lines. ****P < 0.0001.

Even though the neopeptides included in this study were selected by the original study authors based on predicted binding affinity, we asked whether predicted HLA binding could be used to further prioritize the neopeptides. We did this by examining the predicted HLA binding strength of neopeptides and normal peptides with NetMHCpan-4.0 using the EL%Rank score (results for binding affinities are included in Table S1 in Supplementary Material). We found a broad range of predicted binding values of the neopeptides from each study (Figure 1A), and immunogenic neopeptides (neoepitopes) were overall predicted to bind significantly more strongly (*P* < 0.0001, Student’s *t*-test, AUC = 0.72) than non-immunogenic peptides (Figure 1B). Similar but less significant differences were observed when comparing the HLA-binding strength of the immunogenic and non-immunogenic peptides in terms of predicted binding affinity and predicted EL%Rank scores (data not shown). We also analyzed the DAI described by Duan et al. (12), but this was only moderately predictive (AUC = 0.57). The vast majority (75%) of the neoepitopes were predicted to be very strong HLA binders with EL%Rank scores less than 0.5. Only seven neoepitopes had a predicted EL%Rank score greater than 2, and 5 of these were 10- or 11-mer peptides, which all contained nested submer peptides with improved binding to the HLA allele, suggesting that these peptides were not mapped to the minimal epitope (27). The remaining two were 9-mer peptides both containing segments of double or triple cysteines (KVCCCQILL, NLNCCSVPV). Such cysteine-rich peptides are handled poorly by the MHC binding prediction tools due to the bias against cysteines in the peptide data used to train these methods. In fact, replacing the cysteines in the two peptides with X (making NetMHCpan ignore these residues) confirms the strong binding strength (EL%Rank score less than or equal to 1, data not shown). Overall, we hence find that neoepitopes, in accordance with earlier studies analyzing HLA ligands and T cell epitopes in general (9, 11), are characterized by strong predicted binding to the restricting HLA molecules. We find that 96% of the neoepitopes (given the handling of outliers described above) are identified at a EL%Rank threshold of 2.

We plotted the predicted binding strength of neopeptide and normal peptide and observed the previously described pattern (14) that the data can be split into two broad groups (Figure 1C). One group (CB for conserved binding) is defined by peptides where the neo- and normal peptides have comparable binding strength (peptides located close to the diagonal), and one group (IB for improved binding) where the neopeptides have improved binding compared to the normal peptides (peptides located in the upper left corner). As a reflection of the processes applied to selected neo-peptides, very few examples are found where the neopeptide has decreased binding compared to normal.

Next, we split the peptides into two equal sized groups of IB and CB. The split was determined based on the ratio between the EL%Rank scores for the mutated and corresponding normal peptide (EL%Rank_n_/EL%Rank_m_). The IB group included neopeptides, which had at least a 20% improved binding (ratio ≥1.2) whereas the CB group included the remaining peptides (ratio <1.2). Note that this ratio-based measure shares a high overlap with the DAI. As expected, a very large proportion (45%) of the peptides in the IB group are characterized by mutations in the HLA anchor positions, whereas the proportion of peptides with such mutations is low in the CB group (14%) (Figure 1C).

**Figure 2:**
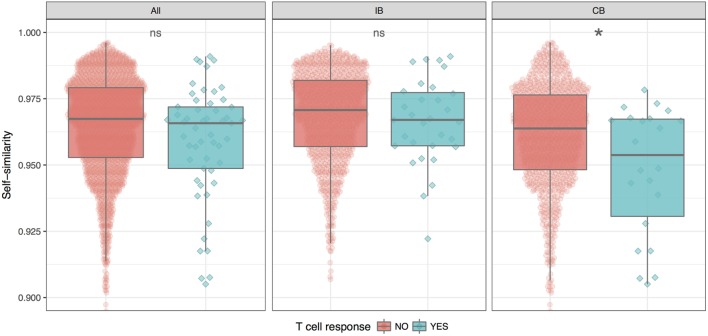
Similarity between neo- and normal peptides. The plot shows the average and SE for the immunogenic (blue) and non-immunogenic (red) peptides for each of the three peptide groups; all (all peptides in the given study), IB (neopeptides with increased binding compared to the normal peptide), and CB (neopeptides with comparable binding compared to the normal peptide). For details, see text. The difference in similarity scores is significant only for CB (*P = 0.025, Student’s *t*-test).

Given the split of peptides into IB and CB, we now investigated how the similarity of the neopeptide to “self” (here taken as the normal counterpart peptide) would impact the peptide immunogenicity. This we did by calculating the similarity between each neo- and normal peptide using the kernel similarity measure proposed by Wen-Jun Shen et al. (27). In short, the similarity in this measure is estimated from the combined set of overlapping kmer (substring peptides of length *k*) peptides. An inherent bias of this approach is that it focuses on the central part of the peptide (for details see Materials and Methods). This bias makes it an ideal first approximation to the HLA fingerprint on T cell interactions with peptide–HLA complexes; here, the C and N terminal positions of the peptide are generally found to play a minor role due to their important contribution to the HLA binding (28). The measure gives a value between 0 and 1 for the similarity of two peptides, where a value of 1 indicates a perfect match. Using this measure, the self-similarity scores of the immunogenic and the non-immunogenic neopeptides were compared for either the complete data set, or the two groups (IB and CB) defined above in terms of the difference in HLA binding strength between mutant and normal peptide (Figure 2).

These results demonstrate that self-similarity in general is a relatively poor predictor for peptide immunogenicity. However, this situation is changed when focusing on the CB subset of peptides where the neo- and normal peptide share comparable HLA binding strength. Here, we found the immunogenic peptides to be significantly less similar to self, compared to the non-immunogenic peptides (*P* = 0.02499, Student’s *t* test). For the IB subset of peptides with improved HLA binding strength of the neopeptide compared to self, immunogenic and non-immunogenic peptides were found to have the same level of self-similarity. The difference in self-similarity score is even more evident when directly comparing IB versus CB peptides among the immunogenic neoepitopes only. Here, we find that CB neoepitopes are indeed characterized by a lower self-similarity score compared to IB neoepitopes (*P* = 0.00395, Student’s *t-*test).

**Figure 3:**
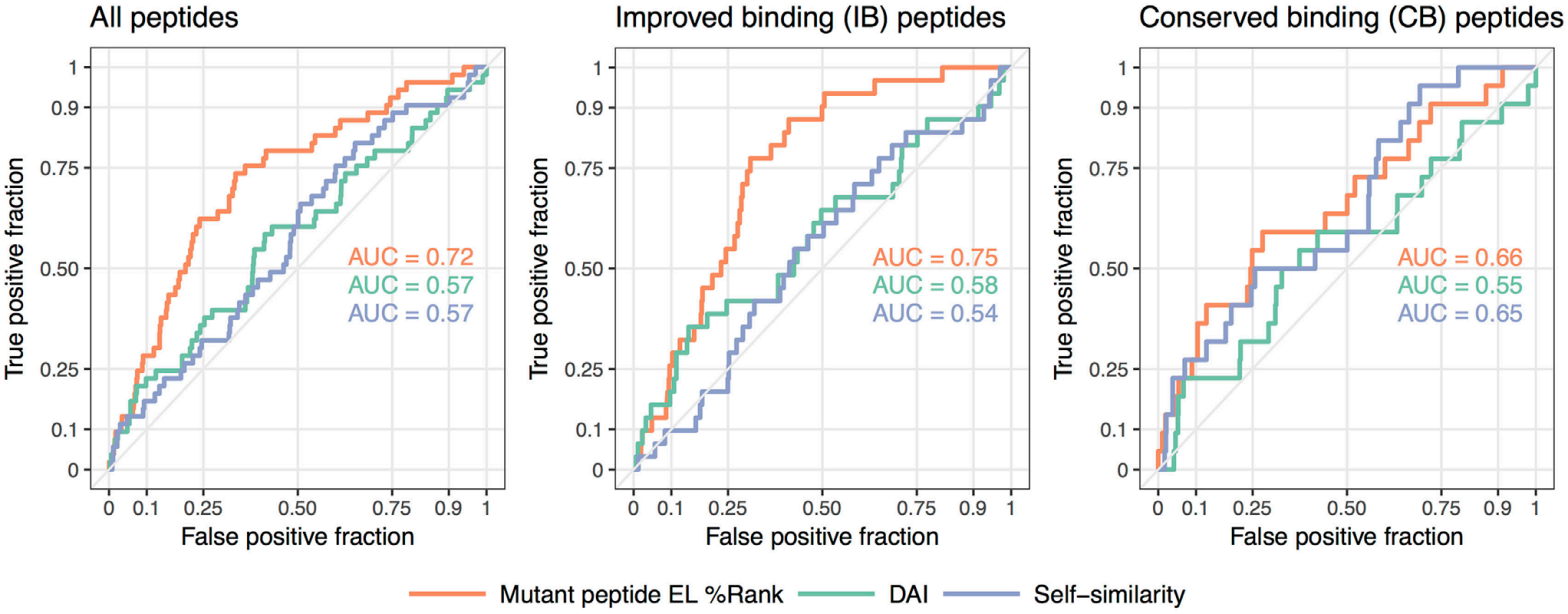
Receiver operator characteristic analyses of the predictive performance of the NetMHCpan-4.0 eluted ligand likelihood percentile rank (EL%Rank) score, the “differential agretopic index” (DAI), and the self-similarity measure. The diagonal line corresponding to AUC = 0.5 is included as a guide to the eye.

We summarize these findings in Figure 3 where receiver operator characteristic (ROC) curves for the predictive performance of HLA binding of the neopeptides, the DAI score, and the self-similarity score are shown for the complete data set and for the two peptide groups IB and CB. In short, a ROC curve is a graphical illustration of the power of a predictive model, in this case, how good predicted HLA binding, DAI and the self-similarity score are at sorting the immunogenic peptides before the non-immunogenic peptides (for details see Materials and Methods). The plots in Figure 3 confirm the above findings, namely that binding strength to HLA is an overall good predictor for neopeptide immunogenicity (the mutant peptide EL%Rank score achieves the highest predictive performance in all 3 plots), that DAI demonstrates poor predictive performance for the data included in the given study (the AUC is low and close to 0.5 in all cases), and that neopeptide self-similarity can be used as an additional correlate besides binding to peptide immunogenicity for peptides with comparable HLA binding between neo- and normal peptide (AUC = 0.65 in Figure 3 CB).

Taken together, these results support the notion that immunogenicity of neopeptides should be predicted using different approaches according to the relationship of the neopeptide HLA binding strength to the binding strength of the counterpart normal peptide. In cases where the neo- and normal peptides both are binders and share similar binding strength, self-similarity plays an important part in the prediction of the neopeptide immunogenicity. This is in contrast to the situation where only the neopeptide is predicted to bind HLA. Here, self-similarity plays a limited role, if any, for the prediction of neopeptide immunogenicity.

## Discussion

Previous studies have analyzed published neoepitopes for patterns in peptide binding affinity (13, 14). To the best of our knowledge, this is the first study analyzing published neoepitopes along with neopeptides from the same studies, which failed to elicit a T cell response. By comparing these two sets of peptides, we identified several patterns that may improve prioritization of candidate neoepitopes.

First, we observed that 9-mer neopeptides were substantially more likely to elicit a T cell response than other peptide lengths. However, it is possible that this reflects the relative inaccuracy in earlier versions of HLA binding prediction algorithms in predicting HLA binding affinity for peptides with length different from 9. Newer versions of, e.g., NetMHCpan better account for peptide length preferences. Indeed, NetMHCpan-4.0 predicts overall stronger affinity for the 9-mers in this study compared to other lengths.

Second, we found that predicted HLA binding was a strong correlate to neopeptide immunogenicity, despite the fact that all neopeptides analyzed in this study were selected in the original publications based on such predictions. Analyzing the HLA binding strength in terms of the EL%Rank score of NetMHCpan-4.0, we found that the immunogenic neopeptide bound HLA significantly stronger than the non-immunogenic peptides. In terms of absolute prediction scores, 96% of the neoepitopes were found to bind with a EL%Rank score of 2 or less. This binding threshold and sensitivity value is in agreement with earlier studies of HLA ligands and T cell epitopes outside the cancer epitope field (9, 11), and suggest that neoepitopes bind HLA with similar binding strength as pathogen derived epitopes. In the context of HLA binding, we also found that the DAI alone is, overall, less predictive than the HLA binding strength of the neopeptide.

Finally, we found that different characteristics were associated with immunogenicity when splitting the peptides into two groups based on neopeptides with conserved binding strength (CB) or improved binding strength (IB) compared to the normal peptide. For the IB neopeptides, no difference in self-similarity between immunogenic and non-immunogenic peptides were observed. In contrast, for the IB neopeptides, the immunogenic peptides were found to share significantly lower self-similarity compared to the non-immunogenic. Given the limitations of the current study (in particular related to the very small number of neoepitopes included), we believe this result to reflect the impact of T cell tolerance on neopeptide immunogenicity. Tolerization against self is only relevant for antigen presented peptides. If a normal peptide fails to bind HLA, no tolerization would have happened against this peptide, and we hence expect similarity toward this peptide to play a minor role in the prediction of immunogenicity. In contrast, we would expect tolerization to take place against a HLA binding self-peptide, and hence also that similarity toward such self-peptides plays a prominent role when predicting neopeptide immunogenicity. This hypothesis is reflected directly in our results.

We are aware that this study suffers from several important limitations. First, 3 of the 13 studies did not provide the peptides, which did not elicit T cell recognition or activation. The non-immunogenic peptides are important for discovering patterns that distinguish non-immunogenic neopeptides from immunogenic neoepitopes. However, a more profound limitation of this study is the small amount of data available. We anticipate that newer high-throughput T cell reactivity screening systems will provide much more data, which will enable a more detailed analysis. Also, it is clear that the model used to assess peptide similarity is very simplistic, and most likely could be refined substantially by for instance taking into account the direct impact on the TCR fingerprint imposed by variations in HLA anchor positions, and by incorporating an amino acid similarity measure different from the protein evolution-based Blosum score used here. Finally, it will be important to evaluate the effects of other factors such as antigen processing, HLA binding stability (29, 30), gene expression, mutant allele frequency, and clonality (23), each of which may be associated with immunogenicity.

## Author Contributions

Design of the study: A-MB, AE, MN, SH. Data collection: A-MB, VJ. Data analysis and interpretation: A-MB, VJ, CB, MN, AE. Drafting the article: A-MB, AE, MN. Critical revision of the article: SH, VJ, CB, ZS. Final approval of the version to be published: A-MB, AE, ZS, SH, MN, CB, VJ.

## Conflict of Interest Statement

The authors declare that the research was conducted in the absence of any commercial or financial relationships that could be construed as a potential conflict of interest. The reviewer ML and handling editor declared their shared affiliation.

## Supplementary Material

Figure S1 Average similarity between peptide pairs with single mutations at different locations within the peptide. The plot was estimated from 3420 single mutant peptide pairs (20 random natural peptides each mutated to 19 single mutant variants at each of the 9 peptide positions).

Table S1 Characteristics of peptides analyzed in this study.
