# Corrigendum: An Analysis of Natural T Cell Responses to Predicted Tumor Neoepitopes

**DOI:** 10.3389/fimmu.2018.01007

**Published:** 2018-05-14

**Authors:** Anne-Mette Bjerregaard, Morten Nielsen, Vanessa Jurtz, Carolina M. Barra, Sine Reker Hadrup, Zoltan Szallasi, Aron Charles Eklund

**Affiliations:** ^1^Department of Bio and Health Informatics, Technical University of Denmark, Kongens Lyngby, Denmark; ^2^Instituto de Investigaciones Biotecnológicas, Universidad Nacional de San Martín, Buenos Aires, Argentina; ^3^Section for Immunology and Vaccinology, National Veterinary Institute, Technical University of Denmark, Kongens Lyngby, Denmark; ^4^Computational Health Informatics Program (CHIP), Boston Children’s Hospital, Harvard Medical School, Boston, MA, United States

**Keywords:** neoepitopes, neoantigens, prediction, immunogenicity, mutations, MHC binding

An outdated version of Supplementary Table 1 was uploaded to the final version of the paper for publication. This table has not been under peer review and does not include the information described in the paper such as the similarity measurement column. The correct Supplementary Table 1 has now been published in the original article. The authors apologize for this oversight. This error does not change the scientific conclusion of the article in any way.

The original article has been updated.

## Conflict of Interest Statement

The authors declare that the research was conducted in the absence of any commercial or financial relationships that could be construed as a potential conflict of interest.

