# Supplementary figures and images for: An Analysis of Natural T Cell Responses to Predicted Tumor Neoepitopes
Source: Front Immunol. 2017 Nov 15;8:1566. doi: 10.3389/fimmu.2017.01566 (PMC5694748; doi:10.3389/fimmu.2017.01566)

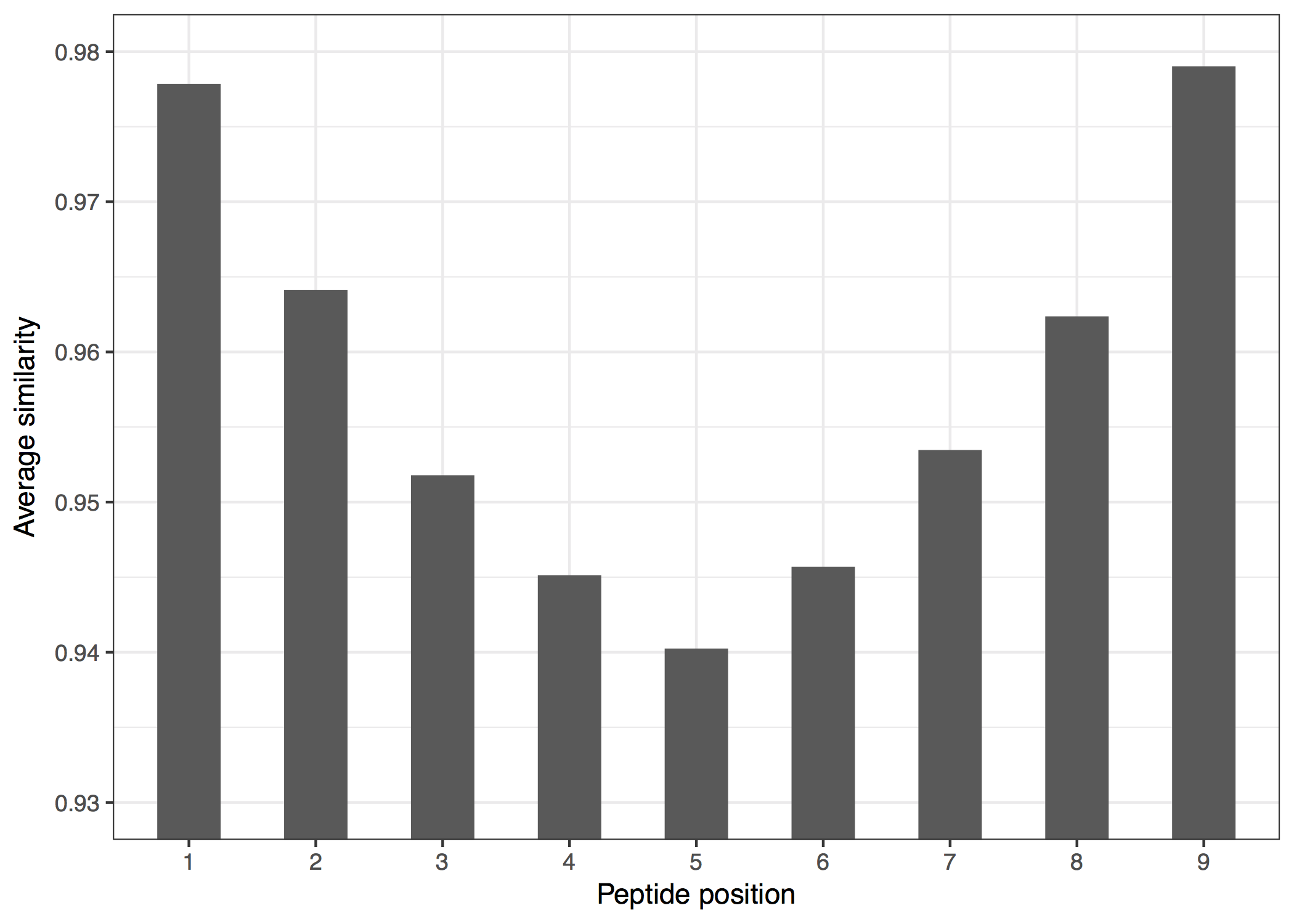

Supplement: Figure S1 — Average similarity between peptide pairs with single mutations at different locations within the peptide. The plot was estimated from 3420 single mutant peptide pairs (20 random natural peptides each mutated to 19 single mutant variants at each of the 9 peptide positions). [file image_1.tiff]
